# Stage-environment fit in high-pressure contexts: differential buffering effects of family support types on adolescent physical activity and sedentary time

**DOI:** 10.3389/fpubh.2026.1788866

**Published:** 2026-04-13

**Authors:** Wenqiong Li, Jie Huang, Hui Jia, Chongyang Han, Dongdong Ma

**Affiliations:** 1 School of Physical Education, Shandong University of Aeronautics and Astronautics, Binzhou, Shandong, China; 2 Faculty of Education and Sports Studies, Universiti Malaysia Sabah, Kota Kinabalu, Sabah, Malaysia; 3 Division of Marine Sports, Pukyong National University, Busan, Republic of Korea; 4 Physical Education Department, Tianjin Medical University, Tianjin, China

**Keywords:** academic stress, accelerometry, adolescence, sedentary behavior, stage-environment fit theory, stress-buffering hypothesis

## Abstract

**Background:**

The transition from middle to high school is characterized by surging academic stress, a precipitous decline in moderate-to-vigorous physical activity (MVPA), and alarming increases in sedentary time (ST). While family support is known to buffer stress, the specific types of support required may shift dramatically across developmental stages. Guided by Stage-Environment Fit Theory and the Stress-Buffering Hypothesis, this study investigated whether the buffering effects of family tangible support versus emotional support on device-measured MVPA and ST differ between middle and high school students.

**Methods:**

A stratified sample of 523 adolescents (298 middle school, 225 high school) from diverse regions in China was recruited. MVPA and ST were objectively measured using ActiGraph GT3X+ accelerometers over seven consecutive days. Self-reported questionnaires assessed chronic academic stress and family support types. Multi-Group Structural Equation Modeling (MG-SEM) was employed to test the moderated effects.

**Results:**

Academic stress robustly predicted decreased MVPA and increased ST across the entire sample. However, the buffering mechanisms exhibited profound developmental heterogeneity. For middle school students, family tangible support significantly buffered the detrimental effects of stress on both MVPA ( β = 0.21, *p* < 0.01) and ST ( β = −0.18, *p* < 0.01). Conversely, for high school students, tangible support became entirely ineffective. Instead, family emotional support emerged as the sole significant buffer against stress-induced physical inactivity ( β = 0.24, *p* < 0.001) and excessive sitting ( β = −0.22, *p* < 0.01).

**Conclusion:**

The efficacy of family interventions is heavily contingent upon developmental timing. Combatting the dual burden of physical inactivity and sedentary behavior in high-pressure educational systems requires a transition from tangible parental involvement in early adolescence to autonomy-respecting emotional support in late adolescence.

## 1 Introduction

Adolescence is a critical epoch for the consolidation of movement behaviors that persist into adulthood. Contemporary public health guidelines unequivocally recommend that adolescents accumulate at least 60 min of moderate-to-vigorous physical activity (MVPA) daily while limiting recreational sedentary time (ST) (1, 2). However, recent global surveillance reveals an ongoing dual pandemic: widespread physical inactivity coupled with pervasive and prolonged sedentary behaviors (3, 4). This crisis is particularly acute in East Asian nations like China, where despite recent educational reforms, the hyper-competitive educational ecosystem continues to place an extraordinary burden on youth (5, 6).

Chronic academic stress has been identified as a primary architect of this behavioral decline. As adolescents navigate the intense pressures of high-stakes testing, structured and unstructured movement opportunities are systematically displaced by extended hours of seated study (7, 8). According to the Stress-Buffering Hypothesis (9), psychosocial support—particularly from the family microsystem—can protect individuals from the pathogenic effects of stressful events. In the context of movement behaviors, a supportive family sports environment can theoretically shield adolescents from stress-induced physical inactivity and excessive sitting (10, 11).

Critically, the existing literature has largely treated “secondary school students” as a homogenous cohort and “family support” as a monolithic construct, masking profound developmental and contextual heterogeneity. According to Eccles’ Stage-Environment Fit Theory (12), optimal adolescent development occurs when the social environment appropriately aligns with their evolving psychological needs. Middle school students and high school students differ fundamentally in their physiological maturation, cognitive independence, and the objective reality of the stressors they face (13). Middle school culminates in the high school entrance examination (*Zhongkao*), a stressor that is often perceived as localized and scaffolded by heavy parental involvement. In contrast, high school culminates in the college entrance examination (*Gaokao*), a distal, pervasive, and chronic stressor that demands immense self-regulation (14).

Consequently, the *type* of family support that effectively buffers stress may shift dramatically. Family support in the physical activity domain can be bifurcated into two primary dimensions: *Tangible Support* (e.g., purchasing sports equipment, driving to facilities, and behavioral co-participation) and *Emotional Support* (e.g., verbal encouragement, understanding, and autonomy support without pressure) (15). For middle school students, direct tangible involvement by parents is often perceived as caring and facilitating. However, for high school students—who endure longer school hours and crave independence—direct parental behavioral involvement might be perceived as overbearing or practically unfeasible, rendering tangible support ineffective. Instead, sophisticated emotional support may become the critical buffer (16, 17).

Despite this strong theoretical premise, empirical studies utilizing objective accelerometry to test the stage-specific buffering effects of distinct family support types remain absent. Addressing this gap is crucial for designing developmentally calibrated public health interventions. Therefore, we formulate three primary hypotheses. First, chronic academic stress will positively predict Sedentary Time and negatively predict MVPA across both cohorts. Second, grounded in Stage-Environment Fit Theory, Tangible Support will significantly buffer the adverse effects of stress on MVPA and ST for middle school students, but not for high school students. Third, Emotional Support will emerge as the dominant, significant buffer against stress for high school students, reflecting a developmental shift toward autonomy-respecting psychological support.

## 2 Methods

### 2.1 Participants and procedure

A multi-stage stratified random sampling design was employed to capture a socioeconomically diverse sample across China. In the initial stage, three provinces representing the Eastern (Shandong province, economically developed), Central (Henan province, developing), and Western (Gansu province, underdeveloped) regions were purposively selected. Subsequently, one middle school and one high school were randomly drawn from each province. Within these schools, intact classes were randomly selected across Grades 7 through 12. Written informed consent was obtained from all participating adolescents and their legal guardians. The protocol was approved by the Institutional Ethics Committee.

Data collection occurred between September and November to avoid seasonal weather extremes. ActiGraph accelerometers and validated questionnaires were distributed to 600 students. After applying stringent wear-time validation criteria and excluding patterned questionnaire responses, the final analytical sample comprised 523 adolescents (effective response rate = 87.2%). To explicitly investigate developmental heterogeneity, this cohort was subsequently divided into two distinct groups based on educational stage: Middle School students (Grades 7–9; *n* = 298, Mean Age = 13.52, SD = 0.84) and High School students (Grades 10–12; *n* = 225, Mean Age = 16.78, SD = 0.91). The gender distribution was balanced (271 males, 252 females).

### 2.2 Measures

#### 2.2.1 Objective MVPA and sedentary time (ST)

Movement behaviors were objectively quantified using ActiGraph GT3X+ triaxial accelerometers (ActiGraph LLC, Pensacola, FL, United States). Participants wore the device on their right hip for seven consecutive days during waking hours. Data were collected at 30 Hz and processed in 60-s epochs using ActiLife 6.0 software. Non-wear time was defined as 60 consecutive minutes of zero counts (18). A valid day required ≥10 h of wear time. Validated Evenson cut-points for adolescents were applied (19): ST was defined as ≤100 counts per minute (CPM), and MVPA was defined as ≥2,296 CPM. Variables extracted were average daily minutes of MVPA and ST.

#### 2.2.2 Academic stress

Chronic educational pressure was measured using the Adolescent Academic Stress Scale (20). This 16-item instrument captures workload, examination anxiety, parental expectations, and peer competition on a 5-point Likert scale (1 = *strongly disagree*, 5 = *strongly agree*). Higher aggregate scores reflect greater perceived chronic stress, and the scale demonstrated excellent internal consistency in the present study (Cronbach’s α = 0.91).

#### 2.2.3 Family support types

Family support was assessed using conceptually distinct subscales adapted from the Family Sports Environment Scale (15). The scale was divided to reflect the theoretical constructs of interest. *Tangible Support* was calculated by aggregating the Physical and Behavioral Environment subscales (15 items; e.g., “My parents provide sports equipment” and “My parents exercise with me”). This subscale showed strong reliability (Cronbach’s α = 0.87). *Emotional Support* was captured using the Psychological Environment subscale (6 items; e.g., “My parents encourage my physical autonomy without pressuring me”). This subscale also demonstrated high internal consistency (Cronbach’s α = 0.85).

### 2.3 Data analysis strategy

Analyses were executed using IBM SPSS Statistics 26.0 and AMOS 24.0. First, preliminary analyses, including descriptive statistics and independent samples *t*-tests, were conducted to ascertain baseline differences between the cohorts. Preliminary assumption checks confirmed that skewness and kurtosis values for all continuous variables were within the acceptable range of ±2, and common method bias was not a pervasive issue. Importantly, because the dependent variables (MVPA and Sedentary Time) were assessed via objective ActiGraph accelerometry rather than self-report, the risk of common method variance inflating the relationships between predictors and outcomes was structurally mitigated. This was further supported by Harman’s single-factor test (variance = 24.6%), justifying the use of maximum likelihood estimation for structural modeling.

Second, a Multi-Group Structural Equation Modeling (MG-SEM) framework was established (21). Latent interaction terms (Stress × Tangible Support; Stress × Emotional Support) were created using the residual-centering approach to circumvent multicollinearity (22). Measurement invariance (configural, metric, and scalar) was confirmed across the two educational stages prior to structural comparisons (23). Model fit was evaluated using $\;\chi^{2}/df$ <3.0, CFI ≥ 0 .90, and RMSEA ≤ 0.08 (24). Critical Ratios for Differences (CRD) were calculated to statistically evaluate whether the buffering pathways significantly differed between middle and high school students.

## 3 Results

### 3.1 Descriptive statistics and cohort differences

As presented in Table 1, the objective data revealed a severe behavioral crisis, particularly in late adolescence. The average daily ST for the high school cohort was alarmingly high (Mean = 615.42 min/day), significantly exceeding the middle school cohort (Mean = 524.38 min/day), t (521) = 11.24, *p* < 0.001. Conversely, high school students accumulated significantly less MVPA (36.03 vs. 47.12 min/day). Academic stress was also significantly elevated in high school ( t = 14.28, *p* < 0.001). Furthermore, while middle school students reported receiving higher levels of Tangible Support, perceptions of Emotional Support remained relatively consistent across stages.

**Table 1:** Descriptive statistics and independent *t*-tests between middle and high school students.

Variable	Total (*N* = 523)	Middle School (*n* = 298)	High school (*n* = 225)	*t*-value	Cohen’s *d*
Academic stress	3.21 (0.75)	2.88 (0.55)	3.65 (0.68)	−14.28***	−1.24
Tangible support	3.48 (0.71)	3.66 (0.65)	3.24 (0.72)	6.94***	0.61
Emotional support	3.58 (0.74)	3.64 (0.70)	3.51 (0.78)	1.99*	0.18
MVPA (min/day)	42.35 (15.62)	47.12 (14.80)	36.03 (14.25)	8.65***	0.76
Sedentary time (min/day)	563.58 (95.4)	524.38 (82.1)	615.42 (88.5)	−11.24***	−1.06

Values are mean (standard deviation). ***p < 0.05, *p < 0.001.

### 3.2 Measurement invariance

Strict scalar invariance was established across the middle and high school cohorts ( $\chi^{2}/df\;$ = 2.24, CFI = 0.952, RMSEA = 0.045, Δ CFI = −0.003 compared to the unconstrained model). This confirmation verified that the constructs were interpreted identically across stages, validating the direct comparison of structural buffering paths.

### 3.3 MG-SEM: the differential buffering effects

**Figure 1:**
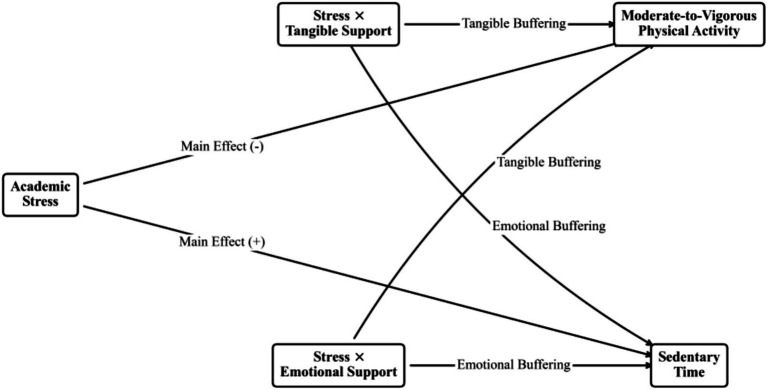
The conceptual multi-group moderated model.

The multi-group structural model testing the dual buffering effects of Tangible and Emotional Support on MVPA and ST demonstrated a good fit ( $\chi^{2}/df$ = 2.38, CFI = 0.941, RMSEA = 0.051) (see Figure 1).

Supporting Hypothesis 1, Academic Stress acted as a robust main effect predictor across both cohorts, significantly increasing Sedentary Time and decreasing MVPA. However, the interactions verifying the Stress-Buffering Hypothesis revealed profound developmental divergence. Table 2 delineates the standardized path coefficients and CRD results.

**Table 2:** Standardized structural path coefficients and critical ratios for differences (CRD).

Structural path	Middle school (*n* = 298)	High school (*n* = 225)	CRD
Main effects			
Stress → MVPA	−0.22***	−0.31***	−1.58
Stress → Sedentary time	0.28***	0.38***	1.62
Buffering interactions (moderation)			
Stress × tangible → MVPA	0.21**	0.04 (ns)	2.54*
Stress × tangible → sedentary time	−0.18**	0.06 (ns)	−2.61**
Stress × emotional → MVPA	0.10 (ns)	0.24***	−2.15*
Stress × emotional → sedentary time	−0.11*	−0.22**	1.98*

*p < 0.05, **p < 0.01, ***p < 0.001. CRD absolute values >1.96 indicate significant path differences.

#### 3.3.1 Middle school: the efficacy of tangible support

**Figure 2:**
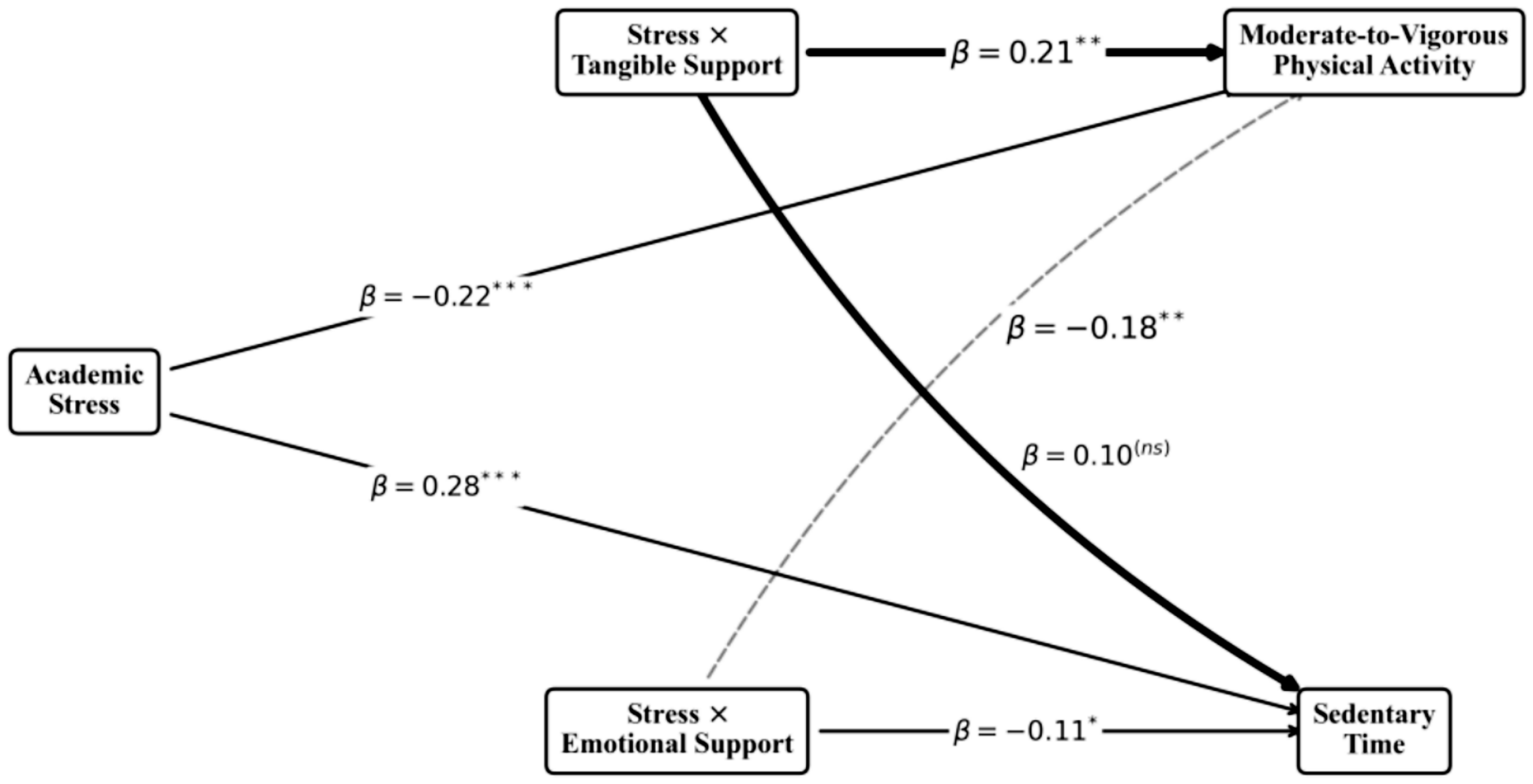
Structural equation model for the high school group.

For early adolescents, Tangible Support was highly effective. The interaction term (Stress × Tangible Support) significantly buffered the negative impact of stress on MVPA ( β = 0.21, *p* < 0.01) and mitigated the stress-induced increase in Sedentary Time ( β = −0.18, *p* < 0.01). Emotional support played a secondary, less prominent role at this stage (see Figure 2).

#### 3.3.2 High school: the shift to emotional support

**Figure 3:**
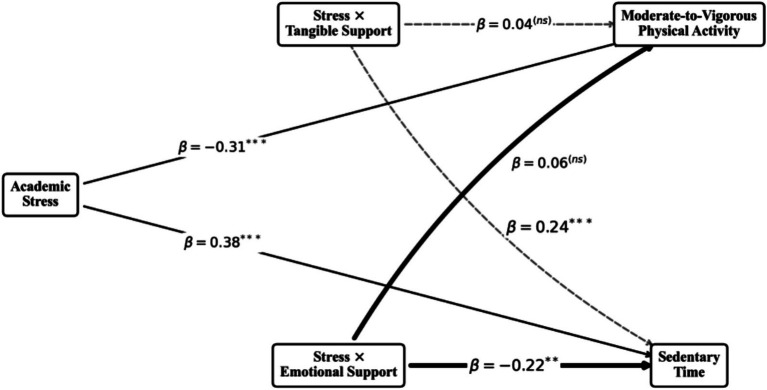
Structural equation model for the middle school group.

For late adolescents facing the *Gaokao*, the buffering mechanism completely inverted. Tangible Support lost all protective efficacy against both physical inactivity ( β = 0.04, *p* > 0.05) and sedentary behavior ( β = 0.06, *p* > 0.05). Instead, Emotional Support emerged as the critical and sole significant buffer. High Emotional Support significantly protected MVPA from stress-induced decay ( β = 0.24, *p* < 0.001) and robustly curtailed the accumulation of Sedentary Time ( β = −0.22, *p* < 0.01). The CRD analysis confirmed that these shifts between support types and educational stages were statistically significant, strongly supporting hypotheses 2 and 3 (see Figure 3).

## 4 Discussion

This investigation deploys a rigorous Multi-Group SEM framework to dismantle the conventional, monolithic understanding of adolescent physical activity and sedentary behaviors. By stratifying the analysis through the lens of Stage-Environment Fit Theory and integrating objective accelerometry, the findings unequivocally demonstrate that the mechanisms governing health behaviors are not static. The stress-buffering role of the family is a developmentally fluid mechanism that must continuously adapt to the evolving psychological maturation and escalating educational stressors of the adolescent. This study extends previous literature by shifting the paradigm from “whether” family support buffers stress to “which type” of support is developmentally appropriate.

### 4.1 The pervasive threat of academic stress on movement Behaviors

Consistent with our first hypothesis, chronic academic stress acted as a universal detriment, robustly predicting decreased MVPA and increased Sedentary Time across both developmental stages. This finding corroborates the “time displacement hypothesis” within behavioral epidemiology, which posits that the demands of high-stakes testing compel adolescents to systematically sacrifice unstructured physical activity in favor of prolonged, seated academic preparation (5, 25).

Beyond mere time constraints, the physiological and cognitive toll of chronic academic stress plays a critical role. The sustained psychological arousal associated with continuous test preparation drains the self-regulatory resources required to initiate physical exertion (7). When cognitive fatigue peaks, adolescents are more likely to default to passive, low-energy sedentary behaviors (e.g., screen time or resting) rather than engaging in physically demanding activities. This dual burden—forced academic sitting and stress-induced physical paralysis—highlights the urgent need for effective psychosocial buffering mechanisms within the adolescent’s immediate microsystem (26).

### 4.2 Early adolescence (middle school): the primacy of tangible support

For middle school students, Tangible Support—manifested through parents purchasing equipment, providing logistical transportation, and engaging in physical activities together—successfully buffered the adverse effects of academic stress. During early adolescence, children are still closely anchored to the family’s physical routine. They typically lack the independent mobility, financial autonomy, or organizational skills required to access structured sports facilities independently (27). Therefore, parents act as the primary “logistical gatekeepers” for health behaviors (10).

When middle schoolers experience stress from the impending *Zhongkao*, they may naturally withdraw from physical exertion. In this context, tangible parental involvement acts as a direct structural scaffold. When a parent explicitly sets up a badminton net or drives the adolescent to a swimming pool, they actively disrupt the adolescent’s sedentary cycle. This active, behavioral environment provides a localized, physical sanctuary from academic pressure. The stress-buffering effect here operates through *instrumental facilitation*—overcoming the barriers of fatigue and stress through direct environmental affordances.

### 4.3 Late adolescence (high school): the critical shift to emotional buffering

The most striking and novel finding of this study is the complete collapse of Tangible Support’s efficacy in high school, superseded by the profound buffering power of Emotional Support. This vividly validates the Stage-Environment Fit Theory (12) and perfectly addresses the developmental heterogeneity often overlooked in previous physical activity research.

As adolescents transition into high school, their psychological architecture undergoes radical changes, characterized by a intensified drive for autonomy, individuation, and peer affiliation (13, 29). Concurrently, the structural reality of the Chinese high school system—which often involves extended evening study sessions, weekend academic classes, and boarding school formats—drastically reduces the physical interface between parent and child (14). Under the crushing, distal pressure of the *Gaokao* (college entrance exam), attempting to exert “Tangible Support” (e.g., a parent trying to force a co-participation run, or excessively monitoring the use of home sports equipment) represents a severe developmental mismatch.

According to Psychological Reactance Theory, when late adolescents perceive parental actions as a threat to their autonomy, they may exhibit oppositional behaviors. In a high-stress state, tangible interventions are often misconstrued by high schoolers as controlling, tone-deaf to their exhaustion, or simply as adding another mandatory “task” to their overloaded schedule. This perfectly explains why tangible support was statistically ineffective in buffering ST or MVPA in the high school cohort.

Instead, Emotional Support—characterized by empathy, validating the adolescent’s stress, and encouraging physical autonomy without exerting pressure—emerges as the essential and sole psychological buffer. High schoolers do not need parents to organize their exercise; they need parents to validate their right to rest and recover. When adolescents perceive their home as an autonomy-supportive haven rather than a secondary academic pressure cooker, they experience a restoration of basic psychological needs (17). This emotional security lowers their allostatic load, enabling them to autonomously utilize physical activity as a functional, internal coping mechanism to dissipate stress, rather than succumbing to stress-induced sedentary paralysis (9).

### 4.4 Practical and policy implications

The findings unequivocally suggest that the “one-size-fits-all” paradigm in family-based health interventions is fundamentally obsolete. Public health guidelines and educational policies must incorporate stage-environment fit principles to be effective.

- For Educational Policy: While the national “Double Reduction” (Shuangjian) policy aims to reduce academic burdens (6, 28), schools must recognize that high schoolers suffer from severe resource depletion. Relying solely on post-school family interventions is inefficient for this group. High schools should mandate daily, high-intensity “micro-breaks” within the curriculum to enforce physical activity, compensating for the limited physical reach of parents.
- For Middle School Families: Interventions should focus on environmental restructuring and parental behavioral co-participation. Parents should be encouraged to invest in accessible home sports equipment and commit to joint weekend physical activities to build strong behavioral habits before the intense high school years begin.
- For High School Families: Intervention efforts must pivot to “Psychological Calibration.” Parents must be educated to step back from behavioral control. The strategy should shift to explicitly framing exercise as a legitimate, guilt-free recovery tool rather than a parental directive. Validating the adolescent’s stress (“You look exhausted from studying, it is completely okay to take a 30-minute walk to clear your head”) is exponentially more effective than rigid logistical scheduling.

### 4.5 Limitations and future directions

While the integration of objective accelerometry and MG-SEM significantly fortifies the rigor of this study, several limitations warrant acknowledgment. First, the cross-sectional design precludes definitive causal inferences regarding the developmental transition. The associations mapped could reflect bidirectional relationships, where physical activity inversely shapes stress appraisals over time. Future research must employ longitudinal, cross-lagged panel models following cohorts across the transition from grade 9 to grade 10. Second, while ActiGraph data eliminates self-report bias and accurately captures volume and intensity, it lacks contextual granularity. It cannot distinguish whether Sedentary Time was accumulated during academic studying or recreational screen time, which may interact differentially with stress. Future protocols should integrate Ecological Momentary Assessment (EMA) alongside accelerometry to capture real-time stress, behavioral contexts, and familial interactions. Finally, future studies should explore how these buffering mechanisms might differ by gender or socioeconomic status within these educational stages.

## 5 Conclusion

The dual burden of physical inactivity and excessive sedentary time among adolescents in high-pressure educational systems is profoundly driven by chronic academic stress. However, families possess the vital capacity to buffer this behavioral decline, provided their support strategies align with the adolescent’s developmental stage. Transitioning from early to late adolescence necessitates a strategic, paradigm-shifting transition from tangible, behavior-oriented parental involvement to sophisticated, autonomy-respecting emotional support. Recognizing, respecting, and implementing this Stage-Environment Fit is absolutely crucial for safeguarding the movement behaviors and long-term psychosocial health of youth.

## Data Availability

The raw data supporting the conclusions of this article will be made available by the authors, without undue reservation.
